# Altruism and its relationship to resilience during disaster

**DOI:** 10.4102/jamba.v18i1.2028

**Published:** 2026-01-23

**Authors:** Bethany L. Van Brown, Brenda K. Vollman

**Affiliations:** 1 Department of Sociology, Criminology and Criminal Justice, Sacred Heart University, Fairfield, United States; 2 Department of Social Sciences, Borough of Manhattan Community College, New York, United States

**Keywords:** disaster, prosocial, altruism, COVID-19, resilience

## Abstract

Studies positively correlate altruism and prosocial behaviour during routine times, yet unexpected environmental triggers (like a disaster) for prosocial and altruistic behaviour are underexplored. People who score highly on the self-reported altruism scale (SRA) are more likely to engage in volunteer activities such as donating blood. What about altruistic and prosocial behaviour during a disaster triggered by natural hazards? What are the characteristics of altruists? Respondents at three different universities were provided with an electronic link via email to complete the survey anonymously, making a total sample size of 182. The data are derived from responses to a longer survey entitled ‘COVID-19 and Risk Perception’. The original purpose of that survey was to explore how perception of risk (to COVID-19) may or may not impact people’s behaviour and included the SRA. Descriptive assessments of all variables were conducted, as well as crosstabs with analysis of variance comparing several means. Data from our study support our hypothesis that people with higher levels of altruism are more likely to engage in pandemic-related helping behaviour. Data also show that the majority of our sample engage in helping behaviour, though the most altruistic and more intrusive (greatest cost) acts were the least common.

**Contribution:**

These are valuable findings for our collective understanding of the nuances of prosocial behaviour. Findings from this study also revealed that people scoring highly on the SRA reported barriers to practising prosocial behaviour. Better understanding these barriers may enable us to eliminate them.

## Introduction

The social scientific study of disaster examines the social processes that make certain groups and communities less able to anticipate, cope with, respond to and recover from disaster (Wisner et al. 2004). In other words, certain demographic groups are more vulnerable to disaster. Decades of disaster research have demonstrated various strains of social vulnerability using the Social Vulnerability Index (Cutter, Boruff & Shirley 2003), and we now know that disasters both reflect the existing social order and may also permanently alter it. For example, more recent research has pointed to enduring mental health challenges among some population segments (Van Landingham 2017), loss of community and intergroup conflict (Erikson 1976), increases in intimate partner violence against women (Enarson 2012), educational disparities among children (and Peek 2011), backlash and discrimination against communities of mixed race (Han, Riddell & Piquero 2022; Peek 2011) and deepening economic inequality (Peacock, Morrow & Gladwin 1997; Tierney 2014).

The question about how humans behave during a disaster is enduring. No answer is absolute, and any one-size-fits-all explanation does not exist, because humans are complex. Thanks to decades of disaster research, we know that human behaviour is impacted by social structure – situational, demographic, environmental, political, economic, cultural, social, psychological and other variables. While we cannot feasibly characterise all human behaviour during a disaster, we can identify behaviour patterns. One of the core areas of inquiry in the social scientific study of disaster is to analyse panic and prosocial behaviour. One of the most common myths about human behaviour during disasters is that people panic and therefore act irrationally. Research has found that many understandable behaviours can be labelled as panic. For example, people not facing an impending threat might still choose to evacuate (Dash & Gladwin 2007). During the beginning of the COVID-19 pandemic, people were highly criticised for panic buying groceries and other household goods such as toilet paper, rather than being applauded (or even recognised) for their preparation efforts (Wachtendorf 2020). These examples, arguably, are normal responses to perceived danger and uncertainty. Decades of disaster research have shown that during the emergency period, people do not typically panic. The much more common – but less newsworthy – behavioural response is to act prosocially. Here, we will conceptualise prosocial behaviour as generally positive behaviour towards others.

Studies positively correlate altruism and prosocial behaviour during routine times (Manzur & Olavarrieta 2021). For example, people who score highly on the self-reported altruism scale (SRA) are more likely to engage in volunteer activities such as donating blood. What about altruistic behaviour during a disaster? Do people who score highly on the SRA engage in more prosocial behaviour? And what are the characteristics of altruistic people? In this study, we set forth to test the hypothesis that those who score highly on the SRA will be more likely to engage in altruistic behaviour. Data from our study about altruism during COVID-19 reveal the characteristics of altruistic people and support the hypothesis that people with higher altruism levels are more likely to engage in pandemic-related helping behaviour. These are valuable findings for our collective understanding of the nuances of prosocial behaviour. Findings from this study also revealed that people who scored highly on the SRA reported barriers to practising prosocial behaviour. Better understanding these barriers may enable us to abolish them.

## Literature review

Social scientists who study disasters focus their attention on the social processes that turn seemingly ‘natural’ hazards into human disasters. In other words, a disaster occurs when a hazard event (hurricane, flood, tornado or pandemic) intersects with the human environment. As Hartman and Squires’ (eds. 2006) title states, “*There is no such thing as a natural disaster: race, class, and Hurricane Katrina*.” Forces of nature, such as hurricanes, tornadoes and floods, can trigger disasters. For example, what follows a flood is a function of the intersection between the hazard, the condition of the built environment and the status of the social structure that shapes the landscape of risk. The social consequences of disaster demonstrate, more broadly, how pre-existing social vulnerabilities relate to one’s ability (or inability) to anticipate, respond to, cope with and recover from disaster.

Researchers did not begin studying disasters systematically in the United States until the late 1940s and early 1950s (Tierney 2019). Social science disaster research accelerated when the US military funded a handful of university-based field research teams to explore how civilians would respond to stressful conditions (Quarantelli 1987). During this time, the impact of wartime stress was the catalyst for our military to support disaster research. Important to note here is that military officials assumed that people would panic, behave in aggressive ways (vs. helping each other) and/or become paralysed with fear. As the number of disaster studies increased, so too did evidence that controverted the assumptions about antisocial human behaviour. Most of the early disaster studies pointed to altruistic, prosocial and highly adaptive behaviour among disaster survivors and first responders (Barton 1969; Fritz 1961; Fritz and Marks 1954; Quarantelli 1966, 1984, 1997; Hoffman 1981; Batson 1990).

Since those early research endeavours, disaster studies have revealed quite a lot about human behaviour in disaster, including applicable information about convergence, panic, crime and conflict and prosocial behaviour, among others. However, myths about human behaviour during disasters prevail. One of the most common and longstanding of these is that people will panic and thus behave irrationally. Decades of empirical research have proven that during the emergency period, people do not typically panic (Quarantelli & Dynes 1977; Dynes and Rodriguez 2005; Rodriguez, Trainor & Quarantelli 2006). The much more common, but less-believed, response is that people help one another.

Many disciplines study prosocial and altruistic behaviour, leading to a lack of conceptual clarity. The differences in the conceptualisations of the two fall along lines of intentions and motives, costs and benefits and the societal context at the time. If we study disaster from a social science perspective, prosocial behaviour is broader and focuses on the mere action itself and refers generally to positive acts towards others (Batson & Powell 2003). Prosocial behaviour, then, can include a wide range of actions intended to benefit one or more people other than oneself. Altruism is more specific and refers to a motivational state related to prosocial behaviour in which the consequences do not play a role. In other words, altruism is a subtype of behaviour with a specific underlying motivation and is not performed with any expectation of rewards. The point is that prosocial behaviour and altruism are distinct but complementary concepts.

Unexpected environmental triggers (like a disaster) for prosocial and altruistic behaviour are underexplored (Pagnini et al. 2025). Existing research shows that helping behaviour and, more specifically, altruistic and prosocial behaviour, can assume many forms during a disaster. For example, helping behaviour may look like neighbours and friends providing shelter, supplies and financial support through their networks (Meyer 2018). Or perhaps, it may manifest when people donate blood and philanthropic trends surge (Meyer et al. 2020). Children and adults may organise donation drives or fundraisers to fulfil unmet needs (Fothergill & Peek 2011). Altruism can be studied in the context of volunteer behaviour when someone helps others for the welfare of those in the community. Spontaneous volunteerism almost always happens and is documented in several previous studies (Kendra & Watchendorf 2016; Lowe & Fothergill 2003; Steffen & Fothergill 2009). Prosocial behaviour was the overwhelming response following Hurricane Katrina (S et al. 2006).

Communities that care for each other, arguably, will be better able to overcome difficulties collectively than people living in individualistic environments. Laguna et al. (2020) add that the perception of the helper affects the act of helping. Notably, helping behaviour is of different types. Amato (1990) delineates formal planned helping behaviour, which is behaviour aimed at helping individuals or groups through agencies or organisations. Informal planned behaviour is aimed at people who are already known and have closeness, such as friends or family. Spontaneous helping behaviour is aimed at strangers, occurs suddenly and is not planned. For example, the altruistic behaviour of community-based volunteers is classified as formal and also informal, planned helping. Kassin, Fein and Marcus (2014) outline three aspects of helping behaviour, namely rewards, empathy and altruism and egoism as a unit. One reason to help is so that the helper experiences the reward of feeling happy when helping others. Empathy aids to help the understanding of someone’s perspective directly or indirectly and the feeling of sympathy for that person. Kassin et al. (2014) argue that altruism and egoism can cause people to perform helping behaviour. Altruistic people want to improve the welfare of others and do not consider the rewards they may receive. People can also perform helping behaviour because of egoism, which is the desire to improve their own well-being. Overall, Kassin et al.’s (2014) findings support that people perform helping behaviour to increase positive feelings within themselves. Our study seeks to better understand who, when and why people might help altruistically when there is no reward.

Solnit (2009) provides historical evidence on earthquakes and hurricanes in the United States and Mexico, illustrating that people reacted with mutual aid and altruism. What is more, across time and place, in times of needs, people become more altruistic (Solnit 2009). Rao et al. (2011) examined prosocial behaviour after the Wenchaun earthquake in China in 2008 and found that survey respondents from the devastated areas gave a larger amount of money and devoted more time to volunteer work versus respondents from non-impacted areas. Apparently, the common experience of disaster can foster shared identity and feelings of mutual support that can make people more willing to help each other (Himma 2010; Pancotto, Giardini & Righi 2024). Experiencing significant material losses can motivate people to become more generous towards others. Research by Jonas (2012), Eränen and Liekind (1993) and Kaniasty and Norris (1995) suggests that post-disaster, people tend to exhibit their best behaviour. Caló-Blanco et al. (2017) found that exposure to an earthquake positively affected several indicators of social cohesion, such as voting and donations to charities, and reduced the crime rate. Cassar et al. (2017) used experimental games to explore trust, risk and time preferences in villages affected and not affected by the 2004 tsunami in Thailand. According to their findings, the tsunami substantially increased prosocial behaviour, and they conclude that natural hazards change people and the communities in which they live – often fostering a sense of community. Indeed, these are interesting findings, and we wish to explore pre-existing levels of altruism and the way they may contribute to civilian disaster response and recovery.

Empathy, or predicting, understanding or experiencing others’ feelings, is an important motivating factor for prosocial behaviour (Batson 2012). Abilities to understand others’ points of view and to feel sympathy and concern for others were found to be strongly associated with altruistic spontaneous behaviours and plans to help, such as charity (Einolf 2008). In other words, the greater someone’s empathy, the higher their propensity for prosocial behaviour (Batson 2012; Dickert, Sagara & Slovic 2011; Einolf 2008).

Earthquakes, floods and other seemingly natural hazards present visible damage, which may or may not be a factor in encouraging altruistic behaviour. What about a pandemic in which the ‘damage’ is less visible – especially when people are quarantined? Some psychological and sociodemographic variables may predict prosocial behaviour during quarantine. Emotional regulation, age and morality may predict prosocial behaviour. Gender-related differences exist as well, in which Auné and Valdés (2024) found that women were more likely to engage in donation and empathetic behaviours. A positive correlation between age and prosocial behaviour was also found. Politically, left-wing tendencies are more associated with the expression of concern for others.

## Research methods and design

### Sampling

We employed a variant of an available sample approach, with a quota sample component to explore our hypothesis. The initial survey was distributed to college students from a largely suburban, residential, private university in Pennsylvania in late spring 2020. This portion of the sample was students enrolled in the College of Arts and Sciences courses, who were provided with an electronic link via email to complete the survey anonymously. Not only was the resultant sample (*N* = 41) small but also three-quarters identified as women and over half as white. In an effort to increase the size and broaden the applicability of results by including a larger male-identified and racially diverse sample, the second wave of the survey was sent to students at two separate Title V^1^ institutions. Respondents were enrolled in introductory criminal justice courses at a large, urban community college in New York, as well as at a large 4-year college in Texas. This wave of surveys was again distributed via an electronic link in late fall 2020. The sample increased nearly over fourfold (N = 142), with less than half identifying as male and over three-quarters as Hispanic or Latino.

Data for this study were stored in a password-protected cloud-based repository. The final responses were downloaded and formatted for SPSS V.27. All categorical and scale responses were coded numerically to allow construction of later-described altruism and helping-behaviour scores, as well as recodes of race.

### Measurement

This study analyses results from survey responses collected electronically. The data are derived from responses to a longer survey entitled ‘COVID-19 and Risk Perception’. The original purpose of that survey was to explore how the perception of risk (of COVID-19) may or may not impact people’s behaviour. The survey comprised 24 questions, 22 of which were constructed as closed ended. Of the closed-ended options, three are multi-select, nine are Likert Scale responses and ten are categorical. The first two scale variables are not included for analysis in this study, as they are health and hygiene related.

### Demographics

The survey included indicators of gender identity (woman, man, genderqueer or nonbinary, agenda and not-specified). Age was included as an open-ended question with a numeric response. Political variables were measured by three questions. Respondents were asked whether they were registered to vote in any state and whether they thought their vote mattered, allowing for yes or no answers. They were also asked how they would describe their views on most political issues, on a five-point scale from very conservative to very liberal.

Race was originally constructed as a multi-select response option, allowing for the broadest classification of this categorical variable while capturing the essence of personal identity construction. No single respondent indicated being American Indian or Alaskan Native or Hawaiian or Pacific Islander alone, while four respondents selected multiple categories. Ultimately, race was recoded, and the four individuals were identified as ‘multiple’. To increase the likelihood of statistical significance in race group comparisons, we again recoded the race variable, collapsing Asian (*N* = 4) with ‘multiple’, creating the ‘multi-other’ category. This approach simplifies analysis, increases the size of subgroups for statistical power, making statistical comparisons more reliable and prevents the exclusion of data for very small groups. Collapsing the race categories also increased the validity of comparative findings for the other racial groups while limiting our ability to generalise to the population at large.

### Scale constructs

We constructed measures indicative of *helping behaviour* and *altruism* by asking several closed-ended questions that were later recoded into these constructs. Included in this paper are the distributions of individual *helping behaviours* and *altruism* measures.

### Altruism

Eleven individual indicators of altruism were measured on a five-point scale of frequency (1–5) in order as follows: never, once, more than once, often and very often. These indicators range in general degrees of intrusiveness, inconvenience, expended energy or ‘cost’. The survey prompt read as follows: please select the category that best reflects the frequency with which you carried out the following, prior to the COVID-19 pandemic. Below are the indicators presented in order of costing the most or encompassing the greatest degree of intrusiveness or inconvenience.

We identified donating blood as the most intrusive, followed by carrying the belongings of a stranger and then volunteering to work at a charity. Two monetary measures are the next most intrusive: giving money to a stranger and giving money to a charity. Measures causing loss of time follow: giving directions to a stranger, delaying the elevator for a stranger or letting someone go ahead in line. A lesser cost is donating clothing and/or goods to charity. This action is followed by helping a classmate with an assignment and, the least intrusive, offering to help the elderly or differently abled.

Additionally, an altruism score was calculated based on the aforementioned behaviour. The altruism score range was 11–33, with a low score measuring low altruism. The conceptual categories for this variable were measured as not altruistic (11–18), altruistic (19–26) and very altruistic (27–33). For this study, the behaviours were not weighted by degree of cost or inconvenience because our analysis includes an indicator of altruism unique to our student-based sample, which we wanted to explore. Additionally, the altruism indicators are affected by the frequency with which the respondent engaged in those activities, which requires a more complex analysis beyond the scope of this descriptive exploration.

### Helping behaviours

Helping behaviour refers to voluntary actions intended to help others. The survey prompt was an individual question defining this before asking, ‘Have you engaged in any of the following helping behaviour since the announcement of the COVID-19 pandemic?’ The response options were seven categorical multi-select indicators. The categories included doing things for ‘someone who qualifies as vulnerable’: grocery shopping, picking up prescriptions and running errands and doing general behaviours, including checking in on family and friends remotely, staying home and obeying government recommendations. In the context of a pandemic, we consider staying home and obeying government regulations to be helping behaviour because they both contribute to community well-being. Staying home reduces exposure to the disease, as well as reducing the likelihood of an everyday emergency (car accident) that could further strain first responders who are already overwhelmed.

A score was calculated by creating a new variable to measure the degree of helpfulness. This method was an additive calculation of the number of helping behaviours that respondents indicated as having engaged in. The survey prompt asked about their engagement in specific behaviours since the COVID-19 pandemic. The score range was 0–7, and the substantive categories were created by collapsing scores as follows: least helping (score 0–1), helping (2–4) and most helping (5–7). Those who engaged in zero or one of the identified helping behaviours were considered least helpful compared to those who engaged in five to seven helping behaviours, being the most helpful.

### Ethical considerations

Ethical clearance to conduct this study was obtained from the Cabrini University’s Institutional Review Board (No. SO-20).

## Results

Initial analyses were descriptive assessments of all variables. Secondary data processing included variable recoding, bivariate crosstabulations and T-tests, with comparisons of means across two or more groups using ANOVA. The bivariate analyses yielded results that established the foundation for this article.

### Demographics

After two waves of data collection, the final sample included responses from 183 students. As noted in Table 1, most often these students were Hispanic or Latino, identifying as women and 25 years of age or younger. The typical age in this sample is 21–23 years old although the respondents have a 34-year range in age (18–52 years old). That said, nearly all (95%) of the sample is 36 years of age or younger.

**TABLE 1:** Sampling characteristics – Altruism and helping in the COVID-19 pandemic (*N* = 183).

Variable	%
Hispanic and/or Latino	63.4
Woman identified	62.4
< 25 years old	82.9
Registered to vote	83.1
Think their vote matters	83.3
Politically moderate	49.4

Other important sampling characteristics are also described here. Most of the sample is registered to vote, thinking that their vote matters and nearly half are politically moderate (Table 1).

In terms of representation, the results of this study may not be generalisable to the overall population in the United States, based on the youthfulness of this sample. However, the size is abundantly sufficient in terms of gender sampling and specific racial categories. On the whole, African Americans are nearly proportionally represented, based on US population data (US Census 2020). Unique to this study the Hispanic (alone), an oft under-represented group, exceeded the total Hispanic representation in the United States more than threefold (Table 2). While our sample under-reflects the Asian (alone) population in the United States, in addition to Whites (US Census 2020), further analysis indicates that these differences seem rooted in the institution type from which the samples were drawn. This finding needs further exploration in our sample and better future outreach when sampling across institution types.

**TABLE 2:** Comparison of racial representation – US population sample response groups.

Racial category	US population (%)†	Study sample (%)‡
Asian people	6.0	3.8
Black/African American people	12.4	10.4
Hispanic/Latino people	18.7	63.4
White/Caucasian people	89.7	18.0

US, United States.

† , United States Census (2020), racial category ‘alone’;

‡ , Survey Administered 2020.

### Altruism

We analysed the response distribution to the eleven indicators of altruism. Of the individual altruistic measures, a majority of respondents engaged in nine of the eleven behaviours and did so *more than once*. Descriptively, over three-quarters of this sample is likely to help a classmate with an assignment (77.3%, Table 3) and offer to help the elderly or differently abled (76.9%). As noted earlier, for the current analysis, the behaviours were not weighted by degree of cost or inconvenience. For this measure, we have previously indicated that these are on the low end of altruism. These behaviours are not as inconvenient or intrusive but still relatively selfless to engage in more than once. Consider that helping a classmate on an assignment, while inconvenient in terms of time and effort, potentially benefits the student offering the assistance. Learning can be augmented when engaged in with others, and spending time with a peer contains a social element. Students helping students may be more broadly prosocial than altruistic in our sample. Additionally, ‘offering’ to help the elderly or differently abled is an idea or intention, but not nearly as inconvenient as actually engaging in the help. No time or effort is lost. Some might also claim that the idea of helping the elderly or differently abled can be considered a social mandate, thereby indicating prosociality rather than altruism.

**TABLE 3:** Descriptive analysis of those who engaged in altruistic behaviours *more than once* (*N* = 183).

Variables – Ordered by the most to the least altruistic level	More than once (%)
Donated blood	25.4
Carried the belongings of a stranger	49.2
Volunteered to work at a charity	56.0
Gave money to a charity	59.3
Gave money to a stranger	61.5
Gave directions to a stranger	63.2
Gave goods and/or clothes to charity	69.2
Delayed the elevator or opened a door for a stranger	71.0
Let someone go ahead in line	74.9
Helped a classmate with an assignment	77.3
Offered to help the elderly or the differently abled	76.9

The behaviours discussed above are closely followed by letting someone go ahead in line at a store (74.9%, Table 3), delaying the elevator or opening a door for a stranger (71%) and giving directions to a stranger (63.2%). These measures involve a potential loss of time, which may be related to a higher regard for the needs of the person receiving the benefit and a selfless willingness to be delayed oneself. The consequence of being late seems to be set aside. Interestingly, a greater proportion of our sample would choose to delay an elevator as opposed to letting someone go ahead in line, though the respondent is still going in the elevator to the same place as the stranger for whom the door was held. Letting someone go in line, especially if there are no other lines opening, involves a greater inconvenience.

We identify a loss of time as less of a personal cost than the next set of altruistic measures, such as spending on or donating goods or money. Just under two-thirds (61.5%, Table 3) of the sample gave money to a stranger more than once, comparable to giving money to charity (59.3%).

While the ordering of indicators from more to less altruistic may not be reflected explicitly in our sample, a majority clearly engaged in most of these behaviours more than once. At least half of our sample was willing to labour for others, volunteer to work at a charity (56%, Table 3) or carry the belongings of a stranger (49.2%). As expected, the most altruistic, and most intrusive act, involving the greatest cost (giving body cells) by donating bloods was least often engaged in more than once. That said, at least one-quarter of the sample did so more than once (25.4%).

Results on the altruism score ranged from 11 to 33. The higher the score, the more the altruism. A descriptive analysis of the score indicates that the central tendency in our sample is very altruistic (26–29, Table 4). Over two-thirds of respondents are very altruistic. The mean is slightly negatively skewed by the small percentage of non-altruistic respondents (10.6%). Just under 3% of the sample earned a score of 11, indicating that the respondent *never engaged in any of the behaviours*; nearly 9% engaged in *all of the behaviours very often* (Table 4). These distributions make sense, given that a majority of respondents engaged in nine of the eleven behaviours and did so more than once (Table 3).

**TABLE 4:** Descriptive analysis of the altruism score (*N* = 179).

Measures of central tendency	Score
**Mean**	26.35
**Median**	28.00
**Mode**	29.00
**Group distributions (%)**	
Not altruistic (score 11–18)	10.60
Altruistic (score 19–26)	29.60
Very altruistic (score 27–33)	69.80
**Score distributions (%)**	
Never engaged in any of the behaviours (score = 11)	2.80
Engaged in all behaviours very often (score = 33)	8.90

### Helping behaviours

For this analysis, we explored the response distribution to the seven indicators of helping behaviours during the pandemic. These are segregated by four behaviours that help others in general, and three focused on helping those considered vulnerable. While we differentiate helping behaviour by the type of individual served, these behaviours are not weighted by the degree of helping related to personal cost or convenience. Of the general helping measures, a majority of respondents (69% – 88%, Table 5) engaged in four of the seven behaviours as follows: just under 90% stayed home during the pandemic, while nearly three-quarters obeyed other government regulations (73%), in addition to isolating and checking in on family and friends remotely (72.7%) and not spreading misinformation (69.4%, Table 5). These distributions could have been anticipated, given that staying at home was required by the government, in addition to masking and abiding by curfews in places like New York City. It might also be considered a given that respondents would check in on family and friends remotely, given the propensity to comply with the government regulations of isolation. For this measure, these helping behaviours are apparently less aligned with altruistic motivation, as they have little cost to the actor.

**TABLE 5:** Descriptive analysis of those who engaged in helping behaviours in the COVID-19 pandemic (*N* = 183).

Variables	More than once (%)
**General helping behaviours**	
Staying home	88.5
Obeying government regulations	73.2
Checking in on family and friends remotely	72.7
Not spreading misinformation	69.4
**Helping those who are vulnerable**	
Running errands	41.4
Grocery shopping	38.3
Picking up prescriptions	31.1

The above findings become especially obvious when we see the relatively dramatic decrease in the sample proportion (only 31% – 41%, Table 5) who engaged in helping someone who was considered a member of vulnerable populations. Interestingly, the earlier analysis of altruistic behaviour revealed that over three-quarters (Table 3) of our sample indicated having offered help to the elderly or differently abled more than once, but that variable was not targeted ‘during the pandemic’. Additionally, ‘offering’ to help the elderly or differently abled is an idea or intention, but not nearly as impactful in actually helping. The idea of helping may also be considered part of the social mandate, consistent with the idea of ‘complying’ with government regulations.

Upon review of the variables representative of those helping the vulnerable, respondents are most likely to run errands (41.1%) and grocery shop (38.3%) for a vulnerable individual, and least likely, of all helping behaviours to pick up prescriptions (31.1%, Table 5). One could argue that to run errands or shop for groceries is not as inconvenient when the respondent is likely to be doing the same for themselves. Most interesting is the lack of help in picking up prescriptions. Those who were extremely vulnerable during the pandemic were also more likely to directly need prescriptions as a result of their vulnerability. One explanation, consistent with the rationale for running errands and grocery shopping, is that these were not inconvenient. Given the youthfulness of the respondents in our sample (Table 1), they are likely not to have been heading to the pharmacy to retrieve their own prescriptions.

Results on the helpfulness score ranged from zero to seven. The higher the score, the more the helpfulness. Descriptively analysing the score indicates that the central tendency in our sample is on the high end of the helpful range (Table 4 and Table 6). Just over half (51.4%) of our sample is considered helping. Our respondents are not at the extremes of this measure although over one-third (38.3%) prove to be most helping as it is defined in this study. Just under 5% earned a score of zero, indicating that the respondent had *not engaged in any of the helping behaviour* since the announcement of the COVID-19 pandemic, while nearly four times that (18%, Table 6) *engaged in all of the behaviours*. It makes sense that, while our sample is very altruistic (Table 4) as measured by the altruism score, the low proportion helping those vulnerable to COVID decreases the ability for our sample to be both very altruistic and very helpful. Had they helped vulnerable populations in greater proportions, our helping score would be higher.

**TABLE 6:** Descriptive analysis of the helpfulness score (*N* = 183).

Measures of central tendency	Score
**Mean**	4.14
**Median**	4.00
**Mode**	4.00
**Group distributions (%)**	
Least helping (score 0–1)	10.40
Helping (score 2–4)	51.40
Most helping (score 5–7)	38.30
**Score distributions (%)**	
Did not engage in any of the behaviours (score = 0)	4.90
Engaged in all behaviours (score = 7)	18.00

## Conclusion

Certainly, a pandemic presents research challenges, especially in collecting data remotely (Kim, Ghimire & Yamashita 2021). A larger sample size is needed to increase statistical significance (Kim et al. 2021). The primary purpose of this paper has been to describe our sample on the measures included in our discussion. This is a jumping-off point from which to explore the comparison of groups, using more complex analyses. Future data analysis will include weighted scales and group comparisons.

Future research can include data from a wider range of respondents to more clearly detect any differences between populations and to better understand the nuances of helping and sharing behaviour during disaster (Kim et al. 2021). Future sampling should include more dedicated outreach to the typically under-represented racial categories overall, particularly Asian and Pacific Islanders in the example of the current study. Additionally, given that age has been shown to predict prosocial behaviour, inclusion of those over 25 years old would allow for better cross-age group analyses. Those groups can be augmented by quota sampling.

As Kim et al. (2021) state:

[*I*]n addition to improved communications and coordination for sharing behaviours during disasters, there is also reason to focus on the sharing behaviours of key demographic groups in key places and times.

which could help disaster researchers better understand helping and prosocial behaviour during disasters. Going forward, we will analyse the engagement in altruistic behaviours, weighted by the degree of cost or inconvenience. Data from our study will allow us to engage in cross-race and gender analyses, in addition to comparative analyses of self-identified politically leaning categories (progressive, conservative and moderate). Actionable items include training programmes that target stakeholders and stakeholder organisations (community-based organisations) to help encourage and support resource sharing within communities during both routine times and disasters (Kim et al. 2021). These training programmes could emphasise resource sharing to support the entire disaster cycle, from preparation to recovery, and resource sharing could also include information on how to overcome various barriers and what successful prosocial behaviour can look like during a disaster (Kim et al. 2021).

## Footnotes

1 *Title V – Developing Hispanic-Serving Institutions Program*. The Developing Hispanic-Serving Institutions (DHSI) Program provides grants to assist HSIs to expand educational opportunities for, and improve the attainment of, Hispanic students. These grants also enable HSIs to expand and enhance their academic offerings, programme quality and institutional stability (US Department of Education 2025).
